# Prediction of small molecule drug-miRNA associations based on GNNs and CNNs

**DOI:** 10.3389/fgene.2023.1201934

**Published:** 2023-05-30

**Authors:** Zheyu Niu, Xin Gao, Zhaozhi Xia, Shuchao Zhao, Hongrui Sun, Heng Wang, Meng Liu, Xiaohan Kong, Chaoqun Ma, Huaqiang Zhu, Hengjun Gao, Qinggong Liu, Faji Yang, Xie Song, Jun Lu, Xu Zhou

**Affiliations:** Department of Hepatobiliary Surgery, Shandong Provincial Hospital Affiliated to Shandong First Medical University, Jinan, China

**Keywords:** small molecule drug, miRNAs, graph neural networks, convolutional neural networks, CNN, liver cancer

## Abstract

MicroRNAs (miRNAs) play a crucial role in various biological processes and human diseases, and are considered as therapeutic targets for small molecules (SMs). Due to the time-consuming and expensive biological experiments required to validate SM-miRNA associations, there is an urgent need to develop new computational models to predict novel SM-miRNA associations. The rapid development of end-to-end deep learning models and the introduction of ensemble learning ideas provide us with new solutions. Based on the idea of ensemble learning, we integrate graph neural networks (GNNs) and convolutional neural networks (CNNs) to propose a miRNA and small molecule association prediction model (GCNNMMA). Firstly, we use GNNs to effectively learn the molecular structure graph data of small molecule drugs, while using CNNs to learn the sequence data of miRNAs. Secondly, since the black-box effect of deep learning models makes them difficult to analyze and interpret, we introduce attention mechanisms to address this issue. Finally, the neural attention mechanism allows the CNNs model to learn the sequence data of miRNAs to determine the weight of sub-sequences in miRNAs, and then predict the association between miRNAs and small molecule drugs. To evaluate the effectiveness of GCNNMMA, we implement two different cross-validation (CV) methods based on two different datasets. Experimental results show that the cross-validation results of GCNNMMA on both datasets are better than those of other comparison models. In a case study, Fluorouracil was found to be associated with five different miRNAs in the top 10 predicted associations, and published experimental literature confirmed that Fluorouracil is a metabolic inhibitor used to treat liver cancer, breast cancer, and other tumors. Therefore, GCNNMMA is an effective tool for mining the relationship between small molecule drugs and miRNAs relevant to diseases.

## Introduction

With the development of sequencing technology, the biomedical field has accumulated a large amount of medical data, which provides more convenience for researchers to study the relationship between diseases and drugs using these data. The prediction of the relationship between small molecule (SM) drugs and microRNAs (miRNAs) has become an important and rapidly developing area in pharmacology and pharmacogenomics research (Bartel, 2004; Beermann et al., 2016; Kozomara et al., 2019; Liu et al., 2022). miRNAs are small non-coding RNA molecules that regulate gene expression and play a key role in various biological processes, including the development of diseases (Cai et al., 2021; Peng et al., 2023). On the other hand, small molecule drugs have been widely used to treat diseases, but their impact on miRNA expression is not clear. However, there are still blind issues in using traditional biological experiments to identify small molecule drug-related miRNAs, which require a lot of experimental time and cost. With the increasing availability of large datasets, it is possible to predict the relationship between small molecule drugs and miRNAs and use this information to improve the efficacy and safety of drugs (Wang et al., 2019; Chen et al., 2020). This field has tremendous potential in discovering new therapeutic targets and developing personalized drugs (Chen et al., 2021; Liu et al., 2023; Xu et al., 2023).

Computational methods have played a crucial role in predicting the association between small molecule drugs and miRNAs (Xu et al., 2020; Zhang et al., 2023). As the available data on drugs and miRNAs continues to increase, various computational methods have been proposed to identify and predict their interactions. Lv et al. (2015) constructed a complete network by combining small molecule similarity networks, miRNA similarity networks, and known small molecule-miRNA association networks. They calculated the similarity of small molecules and miRNAs using a weighted combination strategy, and then used the RWR (Random Walk With Restart) algorithm to predict the potential associations between small molecule drugs and miRNAs. BNNRSMMA first defined a new matrix to represent the small molecule-miRNA heterogenous network using miRNA-miRNA similarity, small molecule-small molecule similarity, and known small molecule-miRNA associations. They then completed this matrix by minimizing its kernel parameter count and used alternating direction multiplication to further minimize the kernel parameter count and obtain prediction scores. They introduced a regularization term to tolerate noise in the integrated similarity. Wang et al. (2022a) proposed a novel dual-network collaborative matrix factorization (DCMF) method for predicting potential SM-miRNA associations. They first preprocessed the missing values in the SM-miRNA association matrix using the WKNKN method, and then constructed a matrix factorization model for the dual network to obtain feature matrices containing potential features of small molecules and miRNAs, respectively. Finally, the predicted SM-miRNA association score matrix was obtained by calculating the inner product of the two feature matrices. Li et al. (2016) proposed a network-based inference model for small molecule-miRNA networks (SMiR-NBI), which relies solely on known SM-miRNA associations. For a given SM, the initial resources are evenly allocated to its associated miRNAs. Then, the resources of each miRNA are allocated to all its associated SMs, and the resources are then redistributed from SMs to their associated miRNAs. The final resources obtained by the miRNAs reflect the likelihood of associations between the given SM and miRNAs. Guan et al. (2018) developed a new graphlet interaction-based inference model for predicting small molecule-miRNA associations (GISMMA). The complex relationships among SMs or miRNAs are described by graphlet interactions, which consist of 28 isomers. The association score for an SM-miRNA pair is calculated by counting the number of graphlet interactions. However, if neither the SM nor the miRNA has a known association, the model cannot predict the SM-miRNA association. Wang et al. (2022b) proposed an ensemble method for predicting small molecule-miRNA associations based on kernel ridge regression (EKRRSMMA). This method combines feature dimension reduction and ensemble learning to reveal potential SM-miRNA associations. Firstly, the authors constructed different feature subsets for SMs and miRNAs. Then, homogeneous base learners were trained on different feature subsets, and the average scores obtained from these base learners were used as the association scores for SM-miRNA pairs. Peng et al. (2022) proposed a new computational method based on deep autoencoder and scalable tree boosting model (DAESTB) to predict the associations between small molecules and miRNAs. Firstly, a high-dimensional feature matrix was constructed by integrating small molecule-small molecule similarity, miRNA-miRNA similarity, and known small molecule-miRNA associations. Secondly, the feature dimension of the integrated matrix was reduced using a deep autoencoder to obtain potential feature representations for each small molecule-miRNA pair. Finally, a scalable tree boosting model was used to predict potential associations between small molecules and miRNAs. Although these models have achieved promising results and played important roles in the development of computational methods for small molecule-miRNA association identification, they have certain issues or limitations: the experimental validation of small molecule-miRNA associations is very limited, and there are many negative associations. When performed on this noisy and sparse small molecule-miRNA association network, the predictors often detect many false negative associations.

**FIGURE 1:**
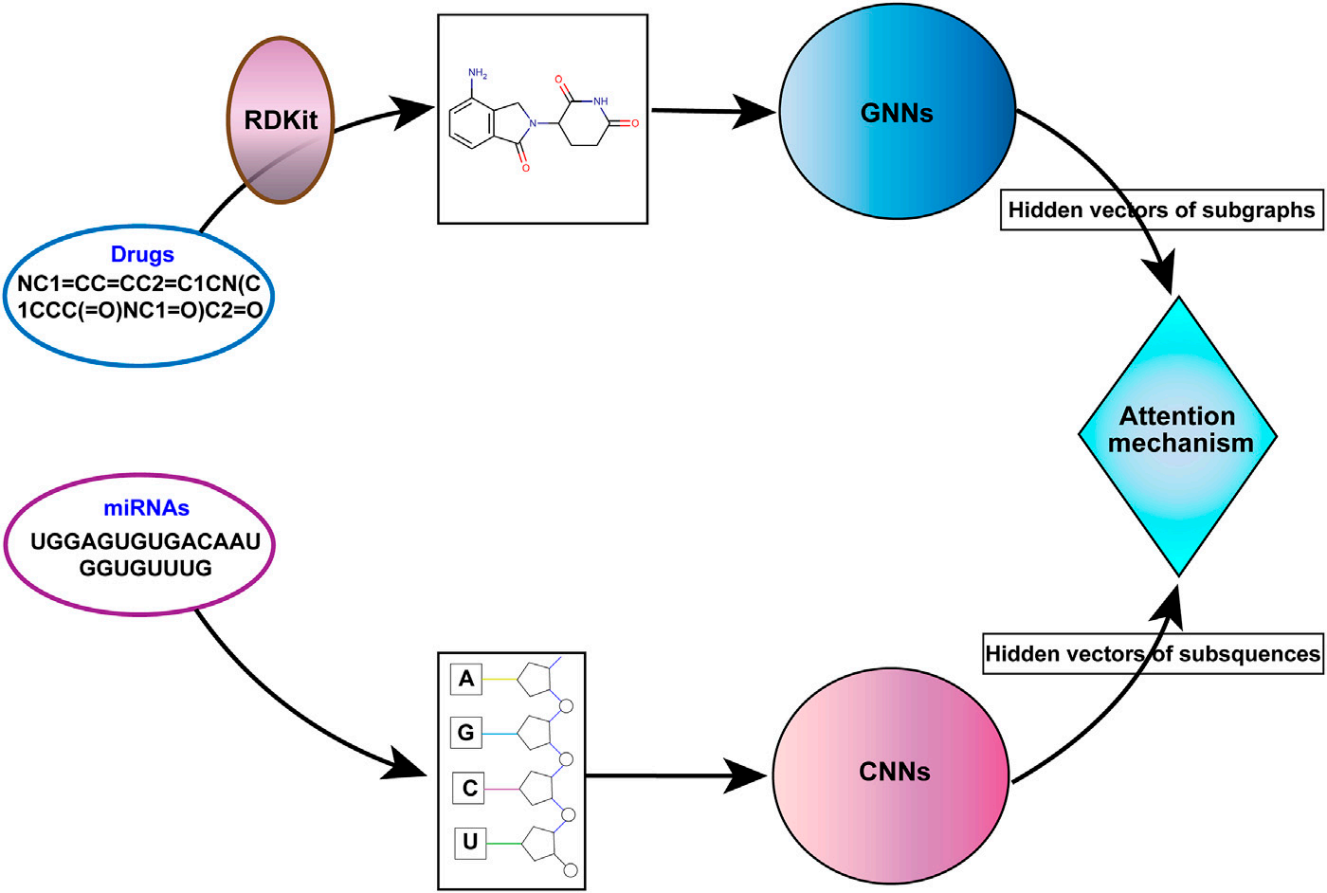
The overall workflow of GCNNMMA.

Therefore, we propose a miRNA-molecule association prediction model (GCNNMMA) by integrating graph convolutional networks (GCNs) (Scarselli et al., 2008) and convolutional neural networks (CNNs) (Chen, 2015) (Figure 1). Firstly, GCNs are used to effectively learn the molecular structural graph data of small molecule drugs, and CNNs are used to learn the sequence data of miRNAs. Due to the black-box nature of deep learning models, it is difficult to analyze and interpret them. Therefore, GCNNMMA introduces a neural attention mechanism (Bahdanau et al., 2014) to address this issue. The neural attention mechanism enables CNNs to learn the weights of sub-sequences in miRNAs, thus predicting the associations between miRNAs and small molecule drugs.

## Materials and methods

### Datasets

For dataset 1, we obtained a total of 664 known small molecule-miRNA associations from SM2miR database (version 1.0) (Liu et al., 2013). Then a total of 831 small molecules were extracted and integrated from SM2miR, DrugBank (Wishart et al., 2018), and PubChem (Kim et al., 2019). 541 miRNAs were collected from SM2miR, HMDD, miR2Disease, and PhenomiR (Ruepp et al., 2010). To evaluate our model performance more comprehensively, we constructed dataset 2, which contains 680 small molecules, 2,460 miRNAs, and 60,212 known small molecule-miRNA associations. Additionally, we downloaded corresponding small molecule drug SMILES data from DrugBank. The SMILES format data was used to describe the spatial structural information of small molecule drugs. Furthermore, we obtained corresponding miRNA sequence data from the miRbase database (Table 1).

**TABLE 1:** Statistics of datasets used in this study.

Dataset	No. of miRNAs	No. of molecules	No. of associations
Dataset 1	541	831	664
Dataset 2	2,460	680	60,212

### Prediction model based on the integration of CNNs and GNNs

#### GNNs process small molecule drug data

**FIGURE 2:**
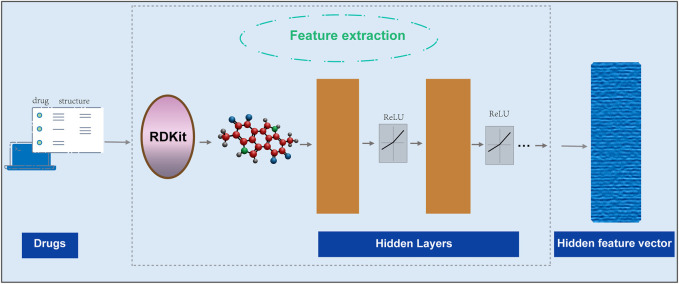
Using GNNs to extract features of small molecule drugs.

End-to-end learning model GNNs has been shown to achieve good performance in many scenarios. Therefore, we first use two functions [the transformation function $tran\left(x\right)$ and the output function $f\left(x\right)$ ] in GNNs to map the molecular structure graph $G\left(V,E\right)$ of small molecule drugs to a low-dimensional vector $y\epsilon R^{d}$ . The transformation function $tran\left(x\right)$ updates the feature information of each node in the molecular graph $G\left(V,E\right)$ using information from neighboring nodes (atoms in the molecular structure graph) and neighboring edges (chemical bonds in the molecular structure graph). The output function $f\left(x\right)$ converts the updated node information in the molecular graph after the transformation function into a low-dimensional vector. In GNNs, both the transformation function and the output function are implemented as differentiable neural networks, and the parameters in the functions are automatically learned through the backpropagation process (Figure 2). The specific steps are as follows:

Subgraph embedding with radius r : Here, we use $G\left(V,E\right)$ to represent a molecular graph, where V is a set of nodes and E is a set of edges. In the molecular structure graph, $v_{i}\epsilon V$ represents the i -th atom and $e_{ij}\epsilon E$ represents the chemical bond between atom i and atom j . Because there are only a few types of nodes (hydrogen and carbon) and edges (double and single bonds) in the molecular graph, representative learning models cannot obtain effective learning results. To solve this problem, GCNNMMA introduces the concept of r -radius subgraphs. An r -radius subgraph describes the set of atoms and chemical bonds within a radius of r with a certain atom as the center. Here, we use $\Gamma \left(i,r\right)$ to represent the set of indices of all adjacent nodes in the subgraph with node i as the center and a radius of r . $\Gamma \left(i,0\right)$ is the node i itself. We use the following definition to describe the subgraph with node $v_{i}$ and a radius of r :

$$\begin{array}{c} v_{i}^{r}=\left(V_{i}^{r},E_{i}^{r}\right) \end{array}$$ (1)

Where, $V_{i}^{r}=\left\{v_{j}|j\epsilon \Gamma \left(i,r\right)\right\}$ , $E_{i}^{r}=\left\{e_{mn}\epsilon E|\left(m,n\right)\epsilon \left(\Gamma \left(i,r\right)\times \Gamma \left(i,r-1\right)\right)\right\}$ Similarly, the subgraph with a radius of r can be defined for the edge $e_{ij}$ : $e_{ij}^{r}=\left(V_{i}^{r-1}\cup V_{j}^{r-1},E_{i}^{r}\cap E_{j}^{r}\right)$ .

Vertex transformation function: In the molecular structure graph G, subgraph embedding can start from any vertex. $v_{i}^{\left(t\right)}\epsilon R^{d}$ is used to describe the vertex i at the t -th step of subgraph embedding information update. The update process is described as follows:

$$\begin{array}{c} v_{i}^{\left(t\right)}=\sigma \left(v_{i}^{\left(t-1\right)}+\underset{j\epsilon \Gamma \left(i\right)}{\sum }h_{ij}^{\left(t\right)}\right) \end{array}$$ (2)

Where $\sigma \left(x\right)=\frac{1}{\left(1+e^{x}\right)}$ , $\Gamma \left(i\right)$ represents the set of neighbor node indices for vertex i . $h_{ij}^{\left(t\right)}$ is a hidden vector describing the information of neighbor node j and the edge $e_{ij}$ between the two nodes for vertex i . It can be calculated using the following formula:

$$\begin{array}{c} h_{ij}^{\left(t\right)}=\max\left(0,W_{neighbor}*\left[\begin{array}{c} v_{j}^{\left(t\right)}\\ e_{ij}^{\left(t\right)} \end{array}\right]+b_{neighbor}\right) \end{array}$$ (3)

Were, $W_{neighbor}\epsilon R^{d\times 2d}$ is a weight matrix and $b_{neighbor}\epsilon R^{d}$ is a bias matrix. $e_{ij}^{\left(t\right)}$ represents the t -th subgraph embedding information update between vertex i and vertex j . By summing the hidden vectors of adjacent nodes and iteratively updating, vertex embedding can gradually learn the global information of the molecular structure graph.

The edge transformation function: The process of updating edge embeddings are similar to the process of updating vertex embeddings. Here, $e_{ij}^{\left(t\right)}$ is used to represent the embedding of the edge between vertex i and vertex j . At the same time, the embeddings of adjacent vertices to the edge, $v_{i}^{\left(t\right)}$ and $v_{j}^{\left(t\right)}$ , are used to update the edge embedding information. The update process is described as follows:

$$\begin{array}{c} e_{ij}^{\left(t\right)}=\sigma \left(e_{ij}^{\left(t-1\right)}+g_{ij}^{\left(t-1\right)}\right) \end{array}$$ (4)

The formula describes $g_{ij}^{\left(t-1\right)}$ as follows: $g_{ij}^{\left(t\right)}=\max\left(0,W_{side}*\left[v_{i}^{\left(t\right)}+v_{j}^{\left(t\right)}\right]+b_{side}\right)$ . $W_{side}\epsilon R^{d\times 2d}$ is a weight matrix, and $b_{side}\epsilon R^{d}$ is a bias vector.

Small molecule output function: To obtain the final output $y_{sm}\epsilon R^{d}$ , the model sums up the embeddings of each vertex in the molecular graph $V=\left\{v_{1}^{\left(t\right)}\left(t\right),v_{2}^{\left(t\right)}\left(t\right),\cdot \;\cdot \;\cdot ,v_{\left|V\right|}^{\left(t\right)}\left(t\right)\right\}$ . The process is described as follows:

$$\begin{array}{c} y_{sm}=\frac{1}{\left|V\right|}\sum_{i=1}^{\left|V\right|}v_{i}^{\left(t\right)} \end{array}$$ (5)

$\left|V\right|$ represents the number of vertices in the molecular graph.

### Using CNNs to process miRNA sequence data

**FIGURE 3:**
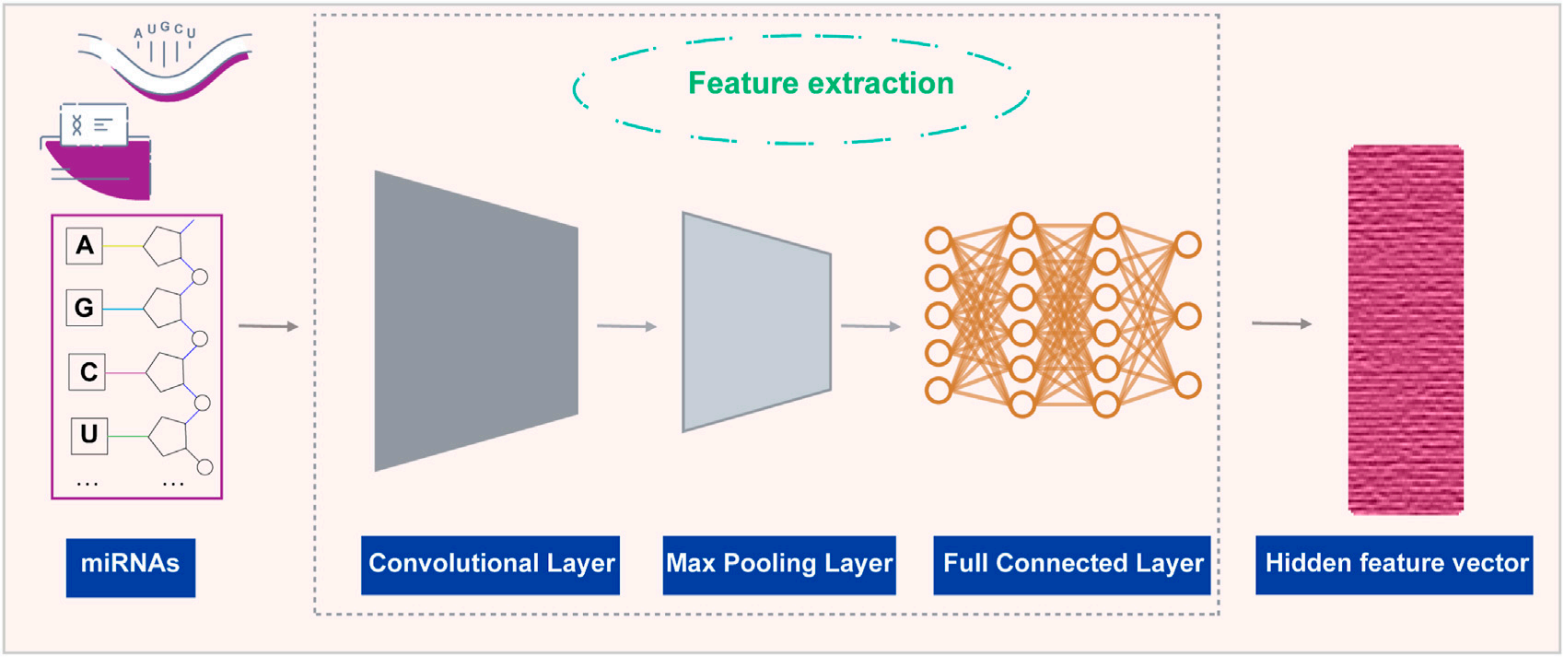
Using CNNs to extract features of miRNAs.

First, CNNs use filter functions to compute a hidden vector $y\in R^{d}$ based on the sub-sequences of the input sequence C and a weight matrix (learned parameters). The filter functions are implemented by neural networks. In CNNs, the overall function $=f\left(C\right)$ is differentiable and all parameters in $f\left(x\right)$ are learned through backpropagation (Figure 3). The specific steps are shown as follows:

#### Sequence input function

To apply CNNs to miRNA sequence data, First, miRNA sequences are defined as “words” consisting of n -length bases (Dong et al., 2006; Costa and De Grave, 2010), where n refers to the number of bases. Then, the miRNA sequence is divided into overlapping n -mers. In this study, to maintain a manageable and informative word vocabulary and to avoid using low-frequency sequence fragmentation in learning representations, a relatively small value of n=3 was set for the number of bases. The miRNA sequence $S=x_{1},x_{2},\cdot \cdot \cdot ,x_{\left|s\right|}$ , where $x_{i}$ is the i -th base pair and $\left|s\right|$ is the length of the sequence, is then split into overlapping n -base pair segments. All words are then translated into randomly initialized embeddings, referred to as “word embeddings.” The word embeddings are ordered as $X_{1},X_{2},\cdot \cdot \cdot ,X_{\left|s\right|-1}X_{\left|s\right|}$ , where $X_{i}\in R^{d}$ is a d -dimensional embedding for the i -th word. Alternatively, we can consider a sequence whose elements consist of concatenated word embeddings. For example, a sequence composed of three consecutive embeddings would be $\left[X_{1};X_{2};\;X_{3}\right],\left[X_{2};\;X_{3};X_{4}\right]\cdot \cdot \cdot \left[X_{\left|S\right|-2};X_{\left|S\right|-1};X_{\left|s\right|}\right]$ , where $\left[X_{i+1};\;X_{i+2};\;X_{i+3}\right]\epsilon R^{3d}$ is the concatenation of $X_{i+1},X_{i+2}$ , and $X_{i+3}$ . Here, $X_{i:i+w-1}$ refers to $\left.{\left[X\right.}_{i};\;\cdot \cdot \cdot ;\;X_{i+w-1}\right]$ , where w is the window size. This processed sequence can be used as input for CNNs.

#### Filter function

Using $X_{i:i+w-1}={\left[X\right.}_{i};X_{i+w-1}]=c_{i}^{\left(0\right)}\epsilon R^{dw}$ as the input to the filter function $f\left(x\right)$ , the output of the filter function is a hidden vector $c_{i}^{\left(1\right)}\epsilon R^{d}$ . The description of the hidden vector is as follows:

$$\begin{array}{c} c_{i}^{\left(1\right)}=f\left(W_{conv}{*c}_{i}^{\left(0\right)}+b_{conv}\right) \end{array}$$ (6)

Where $f\left(x\right)$ is a non-linear activation function, $W_{conv}\epsilon R^{d\times dw}$ is the weight matrix, and $b_{conv}$ is the bias vector. By using the filter function repeatedly, multiple hidden vectors can be obtained:

$$c_{i}^{\left(t\right)}=f\left(W_{conv}*c_{i}^{\left(t-1\right)}+b_{conv}\right)$$ (7)

Multiple hidden vectors form a hidden vector set $C=\left\{c_{1}^{\left(t\right)},c_{2}^{\left(t\right)},c_{3}^{\left(t\right)},......c_{\left|c\right|}^{\left(t\right)}\right\}$ .

miRNA sequence output function. In order to obtain the final output $y_{miRNA}\epsilon R^{d}$ from $C=\left\{c_{1}^{\left(t\right)},c_{2}^{\left(t\right)},c_{3}^{\left(t\right)},......c_{\left|c\right|}^{\left(t\right)}\right\}$ , the average of C is taken. The process is described as follows:

$$\begin{array}{c} y_{miRNA}=\frac{1}{\left|C\right|}\sum_{i=1}^{\left|C\right|}c_{i}^{\left(t\right)} \end{array}$$ (8)

$\left|C\right|$ denotes the number of elements in set C .

### Neural attention mechanism for predicting potential associations between miRNAs and small molecule drugs

GCNNMMA employs a neural attention mechanism to infer interactions between small molecules and subsequences in miRNA sequences. In the collection of hidden vector sequences $C=\left\{c_{1}^{\left(t\right)},c_{2}^{\left(t\right)},c_{3}^{\left(t\right)},......c_{\left|c\right|}^{\left(t\right)}\right\}$ for miRNA sub-sequences, each hidden vector sequence represents its corresponding miRNA sub-sequence. Different miRNA sub-sequences have different binding abilities and probabilities with small molecules. A neural attention mechanism is used to assign corresponding weights to each sub-sequence in the miRNA hidden vector sequence collection, which represents the importance of its association with small molecules. The weight calculation process is described as follows:

$$\begin{array}{c} h_{sm}=f\left(W_{inter}*y_{sm}+b_{inter}\right) \end{array}$$ (9)

$$\begin{array}{c} h_{i}=f\left(W_{inter}*c_{i}+b_{inter}\right) \end{array}$$ (10)

$$\begin{array}{c} \alpha_{i}=\sigma \left(h_{sm}^{T}*h_{i}\right) \end{array}$$ (11)

Where $W_{inter}$ is the weight matrix and $b_{inter}$ _inter is the bias vector. $\alpha_{i}$ represents the strength of interaction between small molecules and miRNA sub-sequences. Based on the calculated attention weights, the final weighted sum can be obtained, as shown below:

$$\begin{array}{c} y_{miRNA}=\sum_{i=1}^{\left|C\right|}\alpha_{i*}h_{i} \end{array}$$ (12)

Finally, the model obtains the final classification output vector $Z\epsilon R^{2}$ by jointly considering $y_{miRNA}$ and $y_{sm}$ :

$$\begin{array}{c} Z=W_{output}*\left[y_{miRNA};y_{sm}\right]+b_{output} \end{array}$$ (13)

Where $W_{output}\in R^{2\times 2d}$ is the weight matrix and $b_{output}\in R^{2}$ is the bias vector. Finally, the output vector $Z=\left[y0,y1\right]$ ] is passed through the softmax function to compute the associated probabilities:

$$\begin{array}{c} P_{t}=\frac{\exp\left(y_{t}\right)}{\sum_{i}y_{i}} \end{array}$$ (14)

## Results

### Performance of GCNNMMA in the cross-validation

**FIGURE 4:**
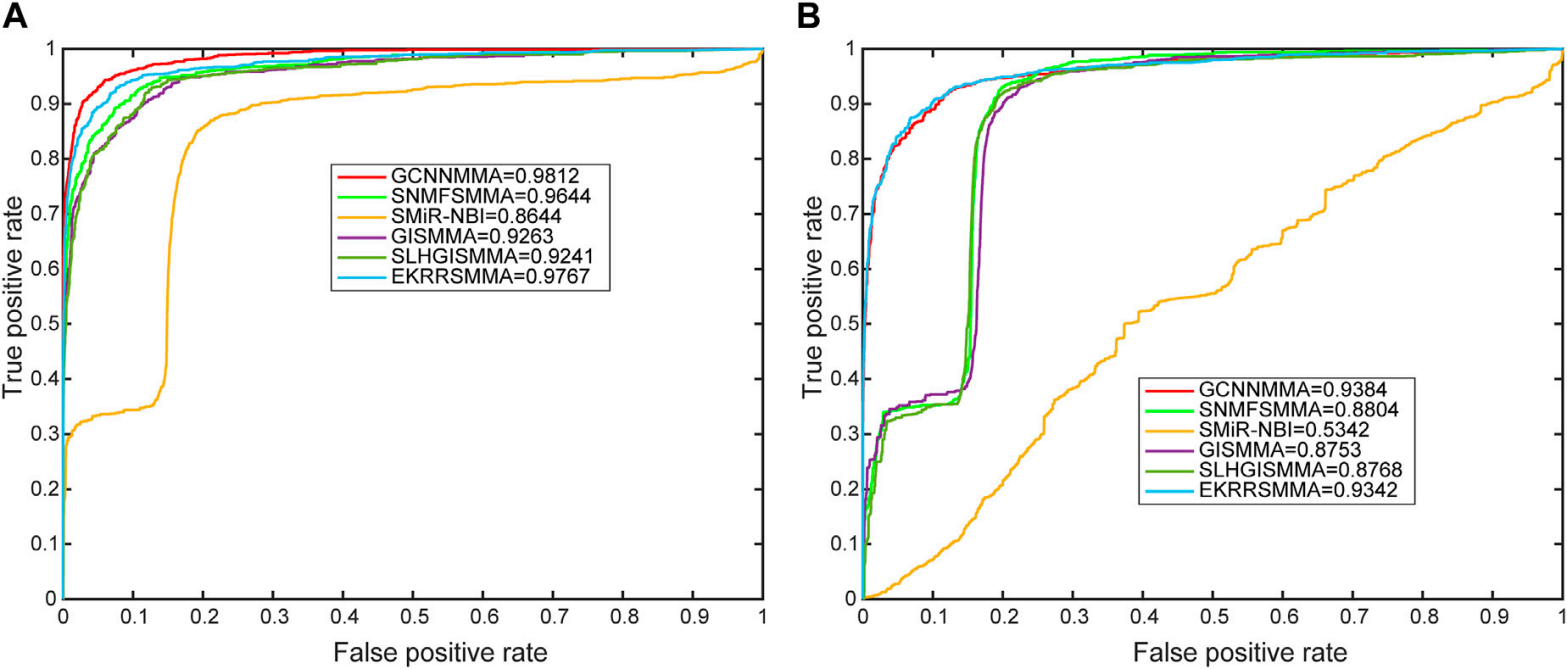
The ROC curves for GCNNMMA and benchmark algorithms for 5-fold CV on the **(A)** dataset 1 and **(B)** dataset 2.

In this work, we compared the performance of the latest five models [SMiR-NBI (Li et al., 2016), GISMMA (Guan et al., 2018), SLHGISMMA (Yin et al., 2019), SNMFSMMA (Zhao et al., 2020), EKRRSMMA (Wang et al., 2022b)] with GCNNMMA, and conducted 5-fold cross-validation (CV) on both dataset 1 and dataset 2 to evaluate the predictive performance of GCNNMMA. All predicted small molecule miRNA pairs were ranked according to the obtained scores. Based on the rankings, we used receiver operating characteristic (ROC) curves to illustrate the performance of our models in the cross-validation runs. As shown in Figure 4, we found that GCNNMMA achieved the best predictive performance on both dataset 1 (AUC = 0.9812) and dataset 2 (AUC = 0.9384). This suggests that GCNNMMA performed the best in predicting the correlation between small molecule drugs and miRNAs.

### GCNNMMA is superior to other popular methods in predicting miRNAs associated with new small molecule drugs

**FIGURE 5:**
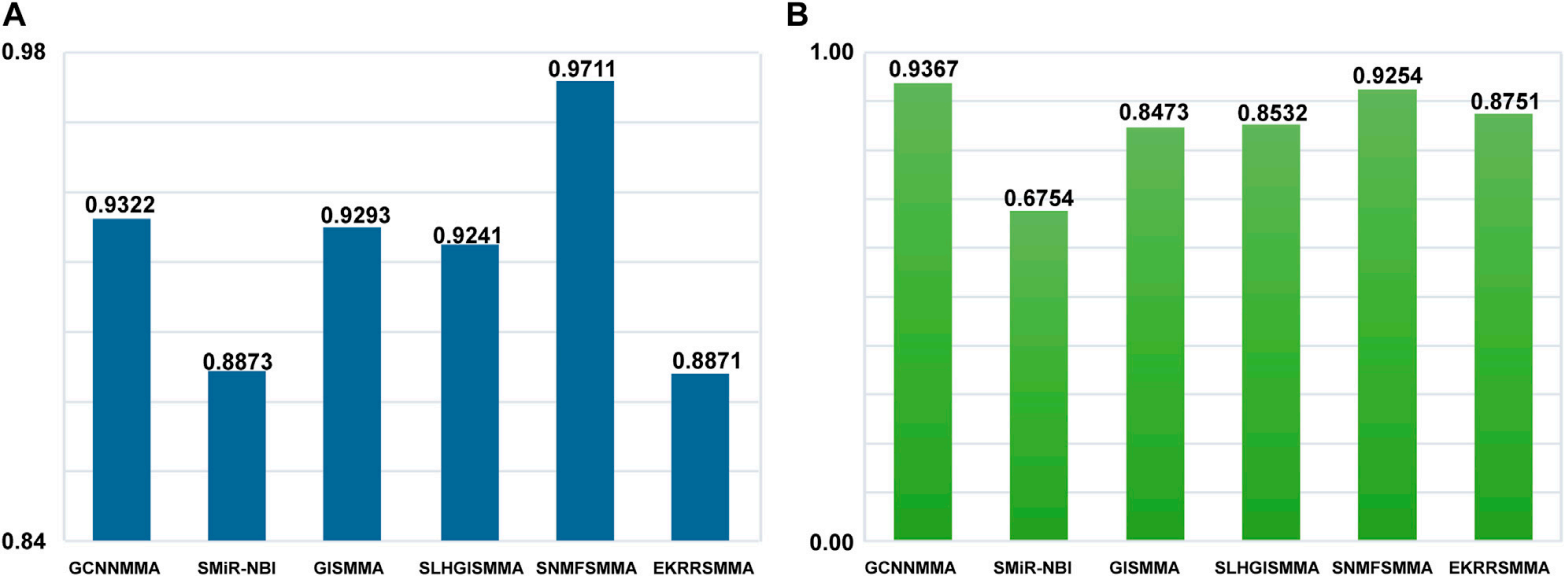
The ROC curves for GCNNMMA and benchmark algorithms for local LOOCV on the **(A)** dataset 1 and **(B)** dataset 2.

It is important to examine the performance of the above method in predicting new miRNAs related to small molecule drugs, in addition to testing the performance of global prediction of small molecule drug-miRNA relationships. A leave-one-out experiment is used to evaluate the ability of the algorithm to predict miRNAs related to new small molecule drugs. To compare the fairness of the test, we still use ROC as the indicator of predictive performance. The local LOOCV experiment was carried on the dataset 1 and dataset 2 (see Figure 5). GCNNMMA showed a higher performance over other approaches in terms of AUC on the dataset 2. Specifically, GCNNMMA obtained AUC value of 0.9367, outperforming that of SMiR-NBI (AUC = 0.6754), GISMMA (AUC = 0.8473), SLHGISMMA (AUC = 0.8532), SNMFSMMA (AUC = 0.9254), EKRRSMMA (AUC = 0.8751). In addition, we can find that the performance of GCNNMMA is also second only to SNMFSMMA on the dataset 1. This also sufficient GCNNMMA is also the best way to predict m miRNAs related to new small molecule drugs.

### Case studies: identifying the relationship between small molecule drugs and miRNAs associated with liver cancer

To further verify the reliability capability of GCNNMMA, we take all known miRNAs-small molecule drug associations in the SM2miR dataset 1 as the training set, and regard the missing miRNAs-small molecule drug associations as candidate sets. After GCNNMMA predicted the interaction probabilities of all candidate miRNAs-small molecule drug associations, we then ranked them according to the predicted probabilities so that the top-ranked associations were most likely to interact. We also validated these top 30 associations by searching for corresponding PubMed literature, as shown in Table 2. Among the top 10, 20, and 30 predicted associations, we were able to validate 6, 12, and 20 associations, respectively through literature search. In the top 10 predicted associations, we found that 5 different miRNAs were associated with Fluorouracil (CID: 3385), a small molecule drug that belongs to the class of pyrimidine analogs and is an anti-metabolic drug used to treat tumors. It interferes with DNA synthesis by blocking the conversion of deoxyuridine monophosphate to thymidine monophosphate (Ellison, 1961). Currently, Fluorouracil is used to treat diseases such as actinic keratosis, breast cancer, colon cancer, pancreatic cancer, gastric cancer, liver cancer, and superficial basal cell carcinoma (Lecluse and Spuls, 2015; Guo et al., 2020). Among the top 20 predicted associations, we discovered novel small molecule drugs associated with miRNAs and Estradiol (CID:5757), Testosterone (CID: 6013), and Dihydrotestosterone (CID: 10635). These three hormones have high bioavailability and can enhance cellular metabolism. These three hormones have high bioavailability and can enhance cellular metabolism (Pentikäinen et al., 2000). Among the top 30 predicted associations, we found that the small molecule drugs Etoposide (CID: 36462) (Wang et al., 2003) and Gemcitabine (CID: 60750) are used for cancer treatment. Etoposide is a semi-synthetic derivative with anti-tumor activity. It inhibits DNA synthesis by forming a complex with topoisomerase II and DNA, inducing double-stranded DNA breaks and preventing repair by blocking the binding of topoisomerase II. Accumulation of DNA breaks prevents cells from entering mitosis, leading to cell death (Uesaka et al., 2007). Gemcitabine (CID: 60750) is a nucleoside analog used in chemotherapy that, like fluorouracil and other pyrimidine analogs, replaces a structural group of nucleic acids in DNA replication to form cytidine in this case. The formation of cytidine stops tumor growth as new nucleosides cannot attach to the “defective” nucleosides, leading to cell apoptosis (cell “suicide”) (Hastak et al., 2010; Vogl et al., 2010). Currently, Gemcitabine is used to treat cancers such as non-small cell lung cancer, pancreatic cancer, bladder cancer, and breast cancer.

**TABLE 2:** Predicting the top 30 small molecule drugs associated with miRNAs.

Rank	CID	miRNA	Evidence (PubMed)	Rank	CID	miRNA	Evidence (PubMed)
1	3,229	hsa-mir-212	28,131,841	16	5,757	hsa-mir-542	17,765,232
2	3,385	hsa-mir-149	27,415,661	17	5,757	hsa-mir-663a	32,215,262
3	3,385	hsa-mir-1915	22,121,083	18	6,013	hsa-mir-135a-1	32,735,753
4	3,385	hsa-mir-203a	25,526,515	19	6,013	hsa-mir-29a	26,296,572
5	3,385	hsa-mir-320a	unconfirmed	20	10,635	hsa-mir-32	20,945,501
6	3,385	hsa-mir-483	unconfirmed	21	10,635	hsa-mir-630	20,945,501
7	3,385	hsa-mir-519c	26,386,386	22	31,401	hsa-mir-603	20,689,055
8	3,385	hsa-mir-617	21,743,970	23	36,462	hsa-mir-26b	31,985,026
9	5,311	hsa-mir-126	unconfirmed	24	36,462	hsa-mir-663a	31,639,426
10	5,311	hsa-mir-409	unconfirmed	25	60,750	hsa-mir-139	33,300,085
11	5,311	hsa-mir-574	unconfirmed	26	60,750	hsa-mir-211	25,789,319
12	5,311	hsa-mir-595	unconfirmed	27	60,750	hsa-mir-299	28,131,841
13	5,311	hsa-mir-744	unconfirmed	28	60,750	hsa-mir-326	unconfirmed
14	5,311	hsa-mir-760	unconfirmed	29	60,953	hsa-mir-137	22,740,910
15	5,757	hsa-mir-17	24,283,290	30	216,239	hsa-mir-664a	unconfirmed

## Discussion

The development of deep learning provides new approaches for predicting the association between small molecule drugs and miRNAs. We developed a prediction model called GCNNMMA based on graph neural networks (GNNs) and convolutional neural networks (CNNs), and validated its performance on two datasets. Experimental results show that GCNNMMA exhibited the best performance in the datasets. Compared with previous similarity-based models, our model extracts the characteristic information of small molecule drugs and miRNAs through GNN and CNN networks, avoiding the dependence on known association information. Furthermore, when predicting the top 30 associations in the dataset, GCNNMMA identified Gemcitabine (CID: 60750) related to hsa-mir-139 and Fluorouracil (CID: 3385) related to hsa-mir-149, both of which are used in cancer treatment by targeting the relevant miRNAs to inhibit cell division and induce cancer cell death. While GCNNMMA achieved good performance, there is still room for improvement, such as integrating multi-source data which remains a challenging problem. In the future, incorporating more data sources, such as miRNA spatial structure data and miRNA precursor data, could improve GCNNMMA. In addition, three-dimensional structural information can better reflect spatial information. One of the future research directions is to utilize the three-dimensional structural information of miRNAs and small molecule drugs to improve prediction accuracy.

## Data Availability

The program and data used in this study are publicly available at: https://github.com/niuzheyu123/GCNNMMA.git.
